# Early Postoperative Increase in Transforming Growth Factor Beta-1 Predicts Microvascular Flap Loss in Reconstructive Surgery: A Prospective Cohort Study

**DOI:** 10.3390/medicina61050863

**Published:** 2025-05-08

**Authors:** Rihards Peteris Rocans, Janis Zarins, Evita Bine, Insana Mahauri, Renars Deksnis, Margarita Citovica, Simona Donina, Sabine Gravelsina, Anda Vilmane, Santa Rasa-Dzelzkaleja, Olegs Sabelnikovs, Biruta Mamaja

**Affiliations:** 1 Intensive Care Clinic, Riga East Clinical University Hospital, Hipokrata Street 2, LV-1079 Riga, Latvia; evitabine@gmail.com; 2 Department of Anaesthesia and Intensive Care, Riga Stradiņš University, Dzirciema Street 16, LV-1007 Riga, Latvia; insana.mahauri@rsu.lv (I.M.); olegs.sabelnikovs@rsu.lv (O.S.); biruta.mamaja@aslimnica.lv (B.M.); 3 Department of Hand and Plastic Surgery, Microsurgery Centre of Latvia, Brivibas Street 410, LV-1024 Riga, Latvia; janis.zarins@mcl.lv; 4 Baltic Biomaterials Centre of Excellence, Riga Technical University, Pulka Street 3, LV-1007 Riga, Latvia; 5 Surgical Oncology Clinic, Riga East Clinical University Hospital, Hipokrata Street 4, LV-1079 Riga, Latvia; renars.deksnis@gmail.com; 6 Laboratory Department, Riga East Clinical University Hospital, Hipokrata Street 2, LV-1079 Riga, Latvia; margarita.citovica@aslimnica.lv; 7 Institute of Microbiology and Virology, Rīga Stradiņš University, Ratsupites Street 5, LV-1067 Riga, Latvia; donsimon@inbox.lv (S.D.); sabine.gravelsina@rsu.lv (S.G.); santa.rasa-dzelzkaleja@rsu.lv (S.R.-D.); 8 Outpatient Department, Riga East Clinical University Hospital, Hipokrata Street 4, LV-1079 Riga, Latvia

**Keywords:** transforming growth factor beta-1 (TGF-β1), TGFB-1, true flap loss, microvascular flap complications, microvascular flap thrombosis, reconstructive surgery

## Abstract

*Background and Objectives:* Microvascular flap surgery is a widely used reconstructive technique for the repair of various defects. Biomarkers have become an essential tool for monitoring flap viability, early detection of complications, and prediction of surgical outcomes. Studies focusing on immunomodulatory cytokines in the early prediction of microvascular flap complications are lacking. We aimed to investigate the predictive value of postoperative changes in transforming growth factor beta-1 (TGF-β1) for microvascular flap complications. *Materials and Methods:* This prospective observational study comprised 44 adults scheduled for elective microvascular flap surgery. Preoperative blood samples for analysis were obtained before surgery, prior to the administration of intravenous fluids. Postoperative blood draws were collected after surgery, before leaving the operating room. Preoperative and postoperative serum concentrations of TGF-β1, as well as preoperative plasma albumin, total protein, total cholesterol, high-density lipoprotein cholesterol, low-density lipoprotein cholesterol, full blood count, albumin, interleukin-6, C-reactive protein, and fibrinogen, were determined. *Results:* Postoperative changes in TGF-β1 were higher in cases with flap loss compared to patients with healthy recovery or patients with minor flap complications (0.403 log10 of ng/mL [0.024–0.782] vs. 0.157 [0.029–0.285] vs. −0.089 [−0.233–0.056], *p* = 0.002). Increased postoperative TGF-β1 was positively linked to preoperative C-reactive protein (*p* = 0.021), fibrinogen (*p* = 0.020), hematocrit (*p* = 0.039), and hemoglobin (*p* = 0.009). *Conclusions:* The postoperative increase in circulating TGF-β1 was associated with microvascular flap complications. Assessment of the postoperative changes in circulating TGF-β1 may be valuable for the early postoperative prediction of true flap loss.

## 1. Introduction

Microvascular flap reconstruction has become increasingly routine due to technological advancements [1], greater surgeon expertise [2], and advances in perioperative care [3]. Biomarkers have become an essential tool in microvascular flap surgery, where they help monitor the viability of tissue flaps, detect complications early, and predict surgical outcomes [3,4]. Recent studies have found multiple preoperative inflammatory [5,6,7] and hemostasis [4,8] biomarkers for the prediction of flap complications, as well as improving the understanding of flap complications’ pathophysiology [3,4]. Microvascular flap surgery is an extensive surgical procedure, which elicits a generally pro-inflammatory immune response [9,10]. Interleukin-6 (IL-6), interleukin-8, and macrophage colony-stimulating factor are pro-inflammatory cytokines that have been linked to ischemia/reperfusion injury in microvascular flap surgery [10]. Pro-inflammatory states have been found to be linked to true flap loss through the modulation of von Willebrand factor [4,8], fibrinogen [8], and platelet function [11]. To date, studies focusing on the role of immunomodulatory cytokines in the pathophysiological patterns of different microvascular flap complications are lacking. Although a previous study used blood samples from microvascular flap blood vessels [10], to date, no studies have evaluated the postoperative changes in immunomodulatory cytokine concentrations in regular circulating blood samples. As an immunoregulatory cytokine, transforming growth factor beta (TGF-β) governs various cellular processes and biological functions, including immune regulation, inflammation, and wound healing [12]. The primary isoform, transforming growth factor beta-1 (TGF-β1) is a promising novel biomarker that influences several processes that can contribute to thrombus formation and stability [13]. TGF-β1 is secreted by platelets and becomes bioactive upon exposure to shear stress [14], which could have pathophysiological and predictive implications in microvascular flap surgery. This study aims to assess the prognostic significance of postoperative TGF-β1 changes in relation to various microvascular flap complications, and to examine the links between perioperative TGF-β1 levels and other biomarkers in microvascular flap surgery patients.

## 2. Materials and Methods

### 2.1. Patient Selection

This prospective observational cohort study included 173 individuals who underwent elective reconstructive microvascular flap surgery at Riga East University Hospital from 1 October 2022 to 31 March 2024. All patients who had true flap loss or secondary flap complications (N = 22) were included in the complications group. To ensure optimal internal validity and comparability between cases over the study period, simple randomization was used [15,16] to select an equal number of patients without complications (N = 22) from the overall cohort. This created a 44-patient cohort to match the available sample count for laboratory analysis. The Riga East University Hospital Science Department (Nr.AP/08-08/22/135) and the Riga Stradins University Ethics Committee (22-2/399/2021) approved the study protocol and the informed consent forms. Adult patients scheduled for elective microvascular flap surgery were included in the study. To avoid confounding factors, patients with coagulation abnormalities, history of smoking, recent blood clotting, or thromboembolic complications [17], along with patients taking hormonal contraceptives or estrogen therapy [18], were excluded, as these patient groups have been associated with flap thrombosis. Patients on anticoagulants or antiplatelet medications were excluded from the study to avoid confounders related to flap bleeding [19]. Patients with active systemic infections [12], or autoimmune disorders [20] were excluded, as these patient groups may have abnormal TGF-β1 levels. Pregnant patients, lactating patients, and children under the age of 18 were also excluded, as they were outside the scope of our study design and were not included in the ethics approval. Patients with incomplete data were also excluded.

### 2.2. Outcome Definitions

True flap loss was defined as compromised blood flow resulting from anastomotic failure or thrombosis that leads to a complete loss of the transposed flap. Minor flap complications were defined as the occurrence of flap infection, delayed or incomplete wound closure, partial flap loss, or wound complications at the harvesting site. All instances of true flap loss required immediate surgical re-exploration.

### 2.3. Perioperative Considerations

The surgical team considered the type of defect, pedicle length, surgical positioning, body mass index, and patient body composition when selecting the flap type. The study included cases with the following flap types: anterolateral thigh, fibular, deep inferior epigastric artery perforator, gracilis muscle, radial forearm, serratus anterior, temporal artery, and latissimus dorsi. All patients received general anesthesia (GA). GA was induced with intravenous administration of fentanyl (1.5 μg/kg), propofol (1–2 mg/kg), and cisatracurium (0.15 mg/kg). All patients were subject to continuous monitoring of electrocardiogram, oxygen saturation, blood pressure (either invasive or noninvasive), body temperature, and end-tidal carbon dioxide levels, starting at the induction of anesthesia. Sevoflurane (Sevorane^®^, AbbVie S.r.l., Campoverde, Italy) at a 0.8–1.2 mean alveolar concentration was used to maintain GA, and fentanyl (1–1.5 μg/kg/h) was used to provide continuous analgesia during surgery. A continuous cisatracurium infusion of 1–2 μg/kg/min was used to achieve intraoperative myorelaxation. Crystalloid fluid administration (RiLac, B. Braun Melsungen AG, Melsungen, Germany) was provided intravenously at a rate of 3.5 to 5.5 mL/kg per hour throughout the surgical and early postoperative periods, with the goal of maintaining urine output at 1–1.5 mL/kg/h. Postoperative monitoring of vitals, temperature, urine output, and pain control was conducted in the recovery unit. All patients received anticoagulation with enoxaparin at a dose of 40 mg daily, commencing on the first postoperative day. The surgical team meticulously monitored the microvascular flap throughout the initial 7 days following surgery. Flap complications were monitored by the surgical team through clinical evaluation of flap perfusion, including assessment of tissue color, temperature, turgor, capillary refill, flap skin texture, the absence of edema, and pinprick tests.

### 2.4. Data Collection, Sample Handling, and Laboratory Analysis

Patient demographics, flap types, surgical indications, recipient sites, operative times, perioperative care, and clinical outcomes were collected from both written and electronic medical records, following a predefined protocol. The surgical outcomes were observed and documented directly by the surgical team. Preoperative blood samples for analysis were obtained immediately before surgery, prior to the administration of intravenous fluids. Postoperative blood draws were obtained after the end of surgery, before leaving the operating room. All blood draws were performed using gentle aspiration and careful handling of the blood collection tubes to avoid artificial stimulation of TGF-β1 release. Full blood count analysis of the preoperative samples was performed using the XN-1500 system (Sysmex Europe SE, Norderstedt, Germany). Preoperative albumin concentrations were obtained using the colorimetric method (Cobas C, Roche/Hitachi, Manheim, Germany). Preoperative IL-6 concentrations were obtained by electrochemiluminescence immunoassay (ECLIA) (Cobas e, Roche/Hitachi, Manheim, Germany). Preoperative C-reactive protein (CRP) concentrations were obtained using the method of immunoturbidimetry (Cobas C, Roche/Hitachi, Manheim, Germany). Preoperative fibrinogen concentrations were obtained using the CS 5100 system (Sysmex Corporation, Kobe, Japan). Preoperative total protein concentrations were analyzed using the colorimetric method (Cobas c, Roche/Hitachi, Manheim, Germany). Preoperative levels of triglycerides, total cholesterol, high-density lipoprotein cholesterol, and low-density lipoprotein cholesterol were measured using the enzymatic colorimetric technique (Cobas c, Roche/Hitachi, Manheim, Germany). All full blood count and clinical chemistry analyses of the preoperative blood samples were processed and analyzed in a clinical laboratory within eight hours of collection. All blood samples designated for TGF-β1 evaluation were frozen within 6 h after collection. Before storage, blood samples for TGF-β1 analysis were centrifuged at 3500× *g* for 10 min. All samples were centrifuged within 2 h after collection. Sample handling was conducted with meticulous care, and light or heat exposure was strictly avoided. The serum samples were preserved at a consistent temperature of −80 °C in screw-cap tubes appropriate for long-term storage. TGF-β1 evaluation was conducted following a single thaw cycle with the TGF-β1 ELISA kit, according to the manufacturer’s protocol (Merck, Darmstadt, Germany). All reagents, calibration standards, and samples were prepared following the instructions outlined in the product’s protocol guide. The assay was conducted at ambient temperature (20–25 °C), in accordance with the manufacturer’s protocol. The absorbance reading was performed on a Varioskan Lux microplate reader (Thermo Fisher Scientific, Waltham, MA, USA) at 450 nm, immediately after the stop solution was added. The acquired measurements were collected for subsequent statistical analysis.

### 2.5. Statistical Analysis

GraphPad Prism, version 5.03 (GraphPad Software Inc., San Diego, CA, USA) and SPSS Statistics for Windows, version 26.0. (IBM Corp., Armonk, NY, USA) were used to perform the statistical analysis. GraphPad Prism version 5.03 (GraphPad Software Inc., San Diego, CA, USA) was used to create graphic visualizations. The distribution of all variables was assessed for normality through visual examination of the quantile–quantile plot. The Kolmogorov–Smirnov test was employed to assess whether the data followed a normal distribution. Analysis of nominal variable datasets was performed using the chi-squared test. Pearson correlation was applied to assess the relationships between parametric datasets. Spearman’s Rho was used to evaluate non-parametric correlations. Differences in data distribution between groups were assessed using the Mann–Whitney U test for non-parametric variables. Independent *t*-tests were conducted to compare the means of two groups with normally distributed data. The datasets for CRP, fibrinogen, hemoglobin, and hematocrit were divided into quartiles. The interquartile differences in mean TGF-β1 concentrations were further compared using the analysis of variance (ANOVA) test. ANOVA test comparisons were also performed for postoperative changes in the log10 of TGF-β1 in different surgical outcome groups. Diagnostic performance was evaluated using the Receiver Operating Characteristic (ROC) curve and the Area Under the Curve (AUC) of postoperative TGF-β1 changes in predicting microvascular flap complications. Cut-off values were determined using the concordance probability method [21]. Odds ratios (OR) for flap complications were calculated using binary logistic regression. Continuous variables with a normal distribution were expressed as the mean with a 95% confidence interval (CI95). Statistical significance was determined at a two-tailed *p*-value of less than 0.05.

## 3. Results

In total, 44 patients were included: 24 (59.1%) men and 20 (40.9%) women. Their mean age was 57.1 years (CI95 52.5–61.7). The complications group consisted of 22 patients, 12 of whom had minor flap complications, while 10 patients had true flap loss. Five of these cases had late flap loss (>72 h). All patients with early true flap loss underwent urgent and successful anastomosis revision. Four cases of late true flap loss were treated with repeated microvascular flap reconstruction, while one case required necrectomy followed by rotated flap reconstruction.

As indicated in Table 1, no significant differences in the rate of flap complications were observed with respect to age, gender, reconstruction sites, surgical indications, or flap types between the control and complications groups. Increased preoperative plasma fibrinogen was found to be associated with flap complications (4.04 [3.56–4.51] vs. 3.22 [2.69–3.75], *p* = 0.044).

When evaluating different indications for surgery, defects had the highest postoperative TGF-β1 concentrations, followed by oncology, while patients with trauma had the lowest preoperative TGF-β1 concentrations (4.25 ng/mL [3.51–4.98] vs. 2.99 [2.51–3.48] vs. 2.33 [1.02–3.64], *p* = 0.023).

No significant differences were found when comparing preoperative TGF-β1, postoperative TGF-β1, or postoperative change in TGF-β1 between the different reconstruction sites or flap types used.

**Figure 1:**
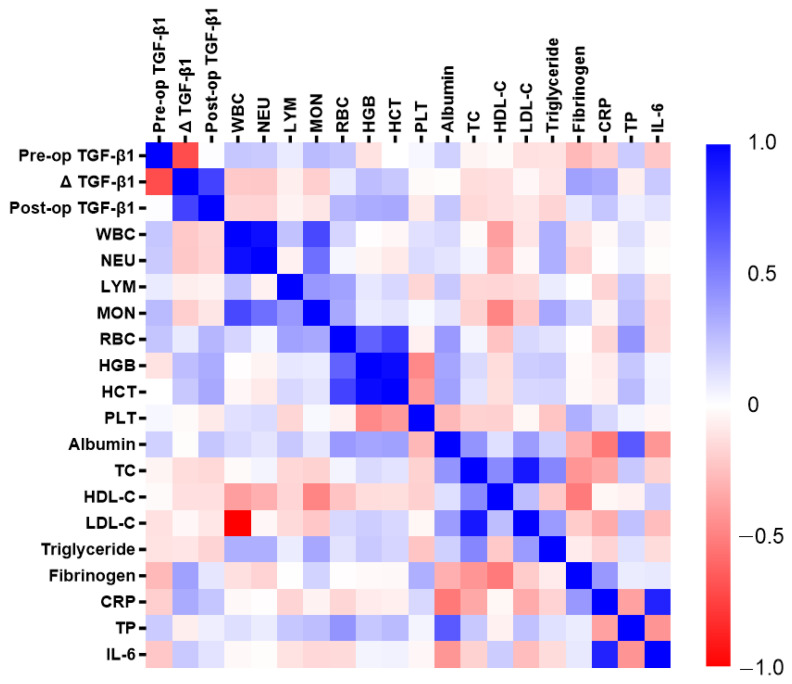
Correlation analysis between transforming growth factor beta-1 and selected biomarkers in microvascular flap surgery patients. Abbreviations—Pre-op (preoperative); TGF-β1 (transforming growth factor beta-1); Δ TGF-β1 (postoperative change in transforming growth factor beta-1); Post-op (postoperative); WBC (white blood cells); NEU (neutrophils); LYM (lymphocytes); MON (monocytes); RBC (red blood cells); HGB (hemoglobin); HCT (hematocrit); PLT (platelets); TC (total cholesterol); HDL-C (high-density lipoprotein cholesterol); LDL-C (low-density lipoprotein cholesterol); CRP (C-reactive protein); TP (total protein); IL-6 (interleukin-6).

As illustrated in Figure 1, postoperative changes in TGF-β1 were positively correlated with preoperative fibrinogen (r = 0.369, *p* = 0.021) and preoperative CRP (r = 0.333, *p* = 0.036). Postoperative TGF-β1 levels were positively correlated with preoperative hemoglobin (*r* = 0.328, *p* = 0.029) and preoperative hematocrit (r = 0.341, *p* = 0.031). There were no significant links between preoperative TGF-β1 and any of the included preoperative biomarkers.

**Figure 2:**
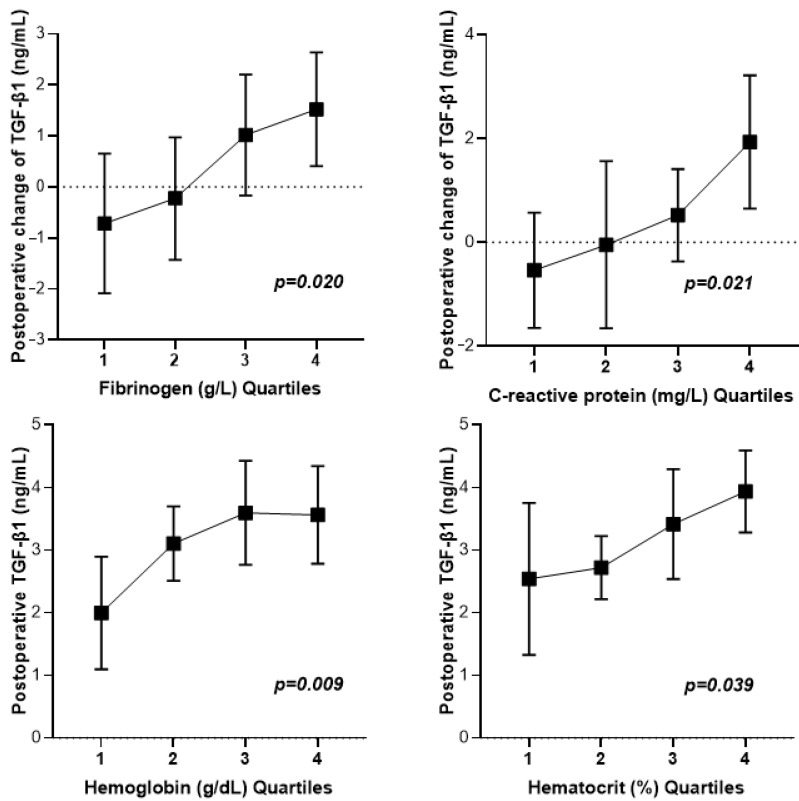
Association between perioperative TGF-β1 and preoperative biomarkers in microvascular flap surgery patients. Quartiles of fibrinogen (g/L): Q1 < 2.31; Q2 2.32–3.78; Q3 3.79–4.34; Q4 > 4.35. Quartiles of C-reactive protein (mg/L): Q1 < 1.93; Q2 1.94–3.86; Q3 3.87–14.00; Q4 > 14.01. Quartiles of hemoglobin (g/dL): Q1 < 11.6; Q2 11.7–12.6; Q3 12.7–13.7; Q4 > 13.8. Quartiles of hematocrit (%): Q1 < 36.6; Q2 36.7–39.0; Q3 39.1–42.2; Q4 > 42.3. Abbreviations—TGF-β1 (transforming growth factor beta-1). Data are presented as the mean (CI95).

As illustrated in Figure 2, postoperative changes in TGF-β1 concentrations were positively associated with preoperative plasma fibrinogen (*p* = 0.020) and plasma CRP (*p* = 0.021). Postoperative TGF-β1 concentrations were positively associated with preoperative hemoglobin (*p* = 0.009) and hematocrit (*p* = 0.039).

**Figure 3:**
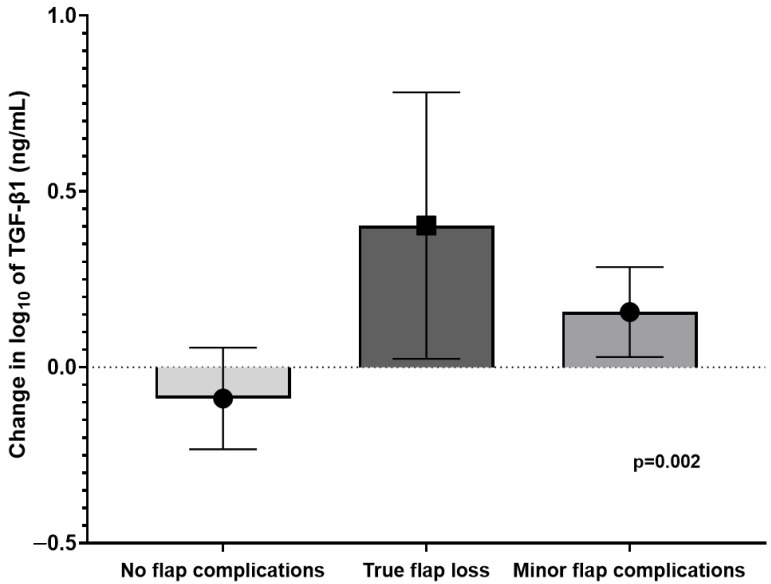
Postoperative changes in log10 of TGF-β1 for different surgical outcomes: ANOVA test comparisons of postoperative changes in log10 of transforming growth factor beta-1 in different surgical outcome groups. Abbreviations—TGF-β1 (transforming growth factor beta-1).

As illustrated in Figure 3, the largest increase in the postoperative log10 of TGF-β1 (ng/mL) was found in cases with true flap loss (0.403 [0.024–0.782]), followed by minor flap complications (0.157 [0.029–0.285]). Patients without flap complications had the lowest postoperative change in the log10 of TGF-β1 (−0.089 [−0.233–0.056], *p* = 0.002). Analysis of the predictive accuracy of postoperative changes in TGF-β1 for true flap loss found that the AUC for log10 of TGF-β1 was 0.797 (0.588–0.997, *p* = 0.005). A postoperative change in TGF-β1 > 1.00 ng/mL was determined to be optimal based on the cut-off analysis (specificity 79.4%, sensitivity 80.0%, positive predictive value 53.3%, negative predictive value 93.1%). When adjusted for age, sex, and preoperative plasma fibrinogen, multivariate regression analysis revealed that an increase in the postoperative change in TGF-β1 increases the odds of true flap loss (OR 2.028, CI95 1.185–3.471, *p* = 0.009).

## 4. Discussion

The central finding of this study is that a postoperative increase in TGF-β1 is linked to true flap loss and, to a smaller extent, minor flap complications. Increased postoperative changes in TGF-β1 are linked to increased CRP and fibrinogen. Increased postoperative TGF-β1 concentrations are linked to increased preoperative hematocrit and hemoglobin.

While TGF-β is secreted by many cell types [22], platelets contribute approximately 45% of the total TGF-β present in plasma [12,23,24], and TGF-β1 is its main isoform, accounting for 95% of the total TGF-β [25]. Previous studies have shown that exposure to shear stress contributes to the release of TGF-β1 from platelets both in vitro [14] and in vivo [14,26]. We propose that the increase in shear forces in a dysfunctional vascular anastomosis site may partially contribute to the postoperative increase in TGF-β1. Prior research indicates that shear stress can efficiently activate latent TGF-β1 present in platelets and the extracellular matrix, highlighting the intricacies of flap survival under dynamic vascular settings [24,26]. In thrombosed arteries, particularly during flap surgery, the augmented shear stress might quadruple, resulting in a synergistic increase in TGF-β1 release [27]. The presence of co-secreted proteins, such as thrombospondin-1, may exacerbate this effect by facilitating the activation of latent TGF-β1 forms [28,29]. Moreover, inflammatory enzymes like matrix metalloproteinases may facilitate this activation process, establishing a feedback loop in which shear-induced TGF-β1 release enhances platelet activation and localized inflammation [28]. Interestingly, increases in TGF-β1 have been found to increase the shear stress exhibited on circulating platelets, and the presence of hypercholesterolemia has been found to exacerbate the release of TGF-β1 from platelets [26], although this was not supported by our findings. It must be noted that the shear stress interaction between the vessel wall and TGF-β1 has been previously studied only in the context of chronic vessel wall changes [26]. In addition to shear-stress-induced TGF-β1 release from platelets, the presence of thrombin during thrombosis of the anastomosis may activate platelets through protease-activated receptors 1/4 (PAR1/4), thereby promoting further TGF-β1 secretion [30]. This process stimulates monocyte tissue factor expression and further amplifies thrombin generation, coagulation, and inflammation [31].

TGF-β1 has also been implicated in the regulation of thrombosis [32,33]. A study by Zhang et al. revealed that the presence of TGF-β1 facilitates the recruitment of neutrophils and monocytes into thrombi while also promoting the formation of neutrophil extracellular traps [13]; therefore, TGF-β1 positively modulates venous thrombus formation. In patients with portal venous thrombosis, increased levels of platelet-derived TGF-β1 have been linked to a hypercoagulable state and contribute to endothelial dysfunction [33]. Notably, the absence of TGF-β1 in mice did not impact the development of arterial thrombosis [13]. However, while our results showed that a postoperative increase in TGF-β1 was linked to true flap loss, we did not observe a link between increased preoperative TGF-β1 and the risk of true flap loss. Our findings corroborate recent evidence that platelet-derived TGF-β1 promotes the progression of venous thrombus development, rather than its initiation [32]. This may imply that the promotion of coagulation is a product of local TGF-β1 release at the site of thrombus formation, rather than its cause.

In venous flap thrombosis, pedicle kinking is a more prevalent cause of flap failure than anastomotic failure [34], and venous thrombosis in such conditions occurs under lower shear stress [35]. In pedicle kinking, blood stasis is considered to be the primary factor for the prothrombotic state at the kinked location [34]. Postoperative increases in TGF-β1 could be predictive of microvascular flap thrombosis progression and imminent flap failure, as the presence of thrombus formation increases the local TGF-β1 concentration [13,36]. This is further supported by our findings that postoperative increases in TGF-β1 were positively linked to increased preoperative plasma fibrinogen, which has also been previously linked to increased rates of flap thrombosis [4,8].

Our results revealed that increased preoperative CRP is positively linked to postoperative increases in TGF-β1. While increased baseline levels of TGF-β1 have been positively linked to CRP in kidney disease patients [37], no previous studies have evaluated the link between CRP and TGF-β1 in a surgical population. Increased preoperative CRP has previously been linked to flap complications [38], which may partially explain our findings.

Our findings indicate that postoperative TGF-β1 concentrations are linked to preoperative hemoglobin and hematocrit. Studies on systemic lupus erythematosus found TGF-β1 concentrations to be positively linked to hemoglobin, although these findings were likely due to lower disease activity [20]. Furthermore, our results did not show any significant link between preoperative hemoglobin and preoperative TGF-β1 concentrations in microvascular surgery patients. A potential explanation for the link between hematocrit and postoperative TGF-β1 is the effect of hematocrit on blood viscosity [39]. Increasing blood viscosity increases shear stress at a given blood flow [40]. As the stimulation of TGF-β1 secretion associated with platelet activation occurs under shear stress [26], increased blood viscosity might increase TGF-β1 secretion from platelets at different stenotic sites in the vasculature [26], and potentially at the site of microvascular anastomosis. It must be noted that increased blood viscosity due to increased hematocrit could also promote TGF-β1 release during the process of blood sample collection [23].

This study has several limitations, as well as several strengths. First, this study includes only a single-center experience and offers a limited patient population due to laboratory resource limitations. The exclusion criteria, which excluded patients with severe comorbidities, pre-existing vascular disorders, or anticoagulant use, may have created selection bias. This could have an impact on the external validity of this study, as the omitted individuals could exhibit substantially varying TGF-β1 concentrations [23]. Conversely, the reported concentrations of TGF-β in humans exhibit considerable variability across both pathological and physiological states, all of which could not be covered by the exclusion criteria [23]. Tracking of TGF-β in plasma may be difficult due to rapid binding to target cells [23]. Therefore, verification with immunohistochemical analysis of SMAD protein expression in target cells would further improve the reliability of the results [23]. Our evaluation of the association between flap loss and TGF-β1 antigen concentrations did not distinguish between arterial and venous thrombotic events at the anastomotic site, despite the potential clinical relevance of this differentiation in relation to TGF-β1 secretion [13]. Given the use of multiple different flap types, the anastomosis site and subsequent vessel curvature were not individually evaluated, although they may influence blood flow and affect outcomes in certain cases. Postoperative TGF-β1 increases may have multiple implications in minor flap complications, such as tissue scarring of the transposed flap [41] or difficult wound healing [42], even after initial flap success. Intriguingly, the emerging technique of platelet-rich plasma injections in reconstructive surgery has demonstrated encouraging outcomes [43]. These benefits may be partially attributed to elevated local concentrations of TGF-β1 [44], although this potential mechanism warrants additional investigation. Further studies with longer postoperative TGF-β1 analysis periods may elucidate potential diagnostic and therapeutic applications of TGF-β1 for scarring and difficult wound healing in microvascular flap surgery.

## 5. Conclusions

The postoperative increase in circulating TGF-β1 is associated with microvascular flap complications. Assessment of the postoperative change in circulating TGF-β1 may be valuable for the prediction of true flap loss. While TGF-β1 has potential as a biomarker for flap viability, it requires improvement in measurement precision and consideration of other factors that impact its activity. Improved understanding of TGF-β1’s dynamics and its clinical implications may lead to better outcomes for patients undergoing microvascular flap surgery.

## Figures and Tables

**Table 1:** General characteristics, perioperative factors, comorbidities, and laboratory parameters: Data are presented as the mean (CI95) or count (percentage). Abbreviations—ENT (ear, nose, and throat surgery); ALT (anterolateral thigh); DIEP (deep inferior epigastric artery perforator); CRP (C-reactive protein); HDL-C (high-density lipoprotein cholesterol); LDL-C (low-density lipoprotein cholesterol); TGF-β1 (transforming growth factor beta-1).

Patient Group	Overall N = 44	Complications Group N = 22	Control Group N = 22	*p*-Value
Demographic data				
Mean age, years	57.9 (54.8–61.0)	59.5 (53.5–65.4)	54.4 (46.7–62.1)	0.389
Women, n (%)	20 (45.5%)	10 (45.5%)	10 (45.5%)	-
Location of reconstruction				
Extremity, n (%)	6 (13.6%)	4 (18.2%)	2 (9.1%)	0.248
ENT, n (%)	26 (59.1%)	14 (63.6%)	12 (54.5%)	0.539
Head and neck, n (%)	6 (13.6%)	2 (9.1%)	4 (18.2%)	0.248
Breast, n (%)	6 (13.6%)	2 (9.1%)	4 (18.2%)	0.248
Flap type				
ALT, n (%)	25 (56.8%)	12 (54.5%)	13 (59.1%)	0.773
Fibular flap, n (%)	5 (11.4%)	3 (13.6%)	2 (9.1%)	0.635
DIEP, n (%)	5 (11.4%)	1 (4.5%)	4 (18.2%)	0.154
Other, n (%)	9 (20.5%)	6 (27.3%)	3 (13.6%)	0.262
Indication				
Trauma, n (%)	5 (11.4%)	2 (9.1%)	3 (13.6%)	0.635
Oncology, n (%)	32 (72.7%)	14 (63.6%)	18 (81.8%)	0.517
Defect, n (%)	7 (15.9%)	6 (27.3%)	1 (4.5%)	0.099
Intraoperative and anesthesia considerations				
Duration of surgery, hours	6.03 (5.48–6.58)	5.93 (5.20–6.66)	6.18 (5.18–7.18)	0.739
Total intraoperative crystalloids, mL	2460.00 (2421.59–2498.41)	2480.00 (2434.76–2525.24)	2440.00 (2370.89–2509.11)	0.276
Total intraoperative colloids, mL	625.00 (521.04–728.94)	650.00 (477.22–822.78)	600.00 (449.19–750.81)	0.615
Intraoperative hematocrit, %	34.50 (33.30–35.70)	33.75 (31.76–35.74)	36.00 (34.16–37.84)	0.097
Use of vasopressors/sympathomimetics, n (%)	15 (34.1%)	10 (45.5%)	5 (22.7%)	0.112
Laboratory values				
Red blood cell count, 10^9^/L	4.13 (3.97–4.28)	4.28 (4.06–4.49)	4.00 (3.77–4.23)	0.084
White blood cell count, 10^9^/L	6.36 (5.57–7.17)	6.50 (5.62–7.38)	6.25 (4.91–7.59)	0.418
Lymphocyte count, 10^9^/L	1.67 (1.48–1.86)	1.63 (1.31–1.93)	1.71 (1.45–1.96)	0.497
Neutrophil count, 10^9^/L	3.90 (3.13–4.67)	4.06 (3.28–4.84)	3.76 (2.44–5.09)	0.162
Monocyte count, 10^9^/L	0.56 (0.50–0.63)	0.56 (0.47–0.66)	0.57 (0.47–0.66)	0.958
Platelet count, 10^9^/L	258.95 (230.52–287.38)	288.37 (238.33–338.40)	233.55 (202.96–264.14)	0.092
Hemoglobin, g/dL	12.43 (11.90–12.95)	12.87 (12.13–13.61)	12.05 (11.28–12.81)	0.087
Hematocrit, %	38.81 (37.41–40.22)	40.12 (38.22–42.01)	37.69 (35.64–39.74)	0.065
Total plasma protein, g/L	63.79 (61.94–65.94)	64.08 (61.32–66.83)	63.51 (60.79–66.23)	0.794
Plasma albumin, g/L	38.77 (37.58–39.95)	39.00 (37.05–40.96)	38.55 (36.98–40.11)	0.668
CRP, mg/L	8.40 (3.90–12.91)	6.93 (3.25–10.61)	9.87 (1.23–18.51)	0.718
Plasma fibrinogen, g/L	3.61 (3.24–3.98)	4.04 (3.56–4.51)	3.22 (2.69–3.75)	0.044
Interleukin-6, pg/mL	14.62 (10.32–18.92)	11.73 (6.32–17.13)	17.37 (10.51–24.24)	0.262
HDL-C, mmol/l	1.27 (1.16–1.39)	1.17 (1.01–1.32)	1.37 (1.20–1.54)	0.094
LDL-C, mmol/l	2.89 (2.57–3.21)	2.84 (2.49–3.19)	2.93 (2.38–3.49)	0.950
Preoperative TGF-β1, ng/ml	2.64 (2.25–3.03)	2.68 (2.13–3.24)	2.60 (2.01–3.20)	0.771
Postoperative TGF-β1, ng/ml	3.12 (2.71–3.53)	3.48 (2.90–4.06)	2.77 (2.20–3.35)	0.072

## Data Availability

The corresponding author will provide access to the analyzed datasets upon reasonable request. The corresponding author is responsible for securing the protection of individual privacy when transferring the datasets.
